# Death of Prof. Swett

**Published:** 1854-11

**Authors:** 


*Death of Prof. Swett.*—Dr. John A. Swett, Professor of Practice in the Medical Department of the New York University, died a few weeks since. The death of Prof. Swett is a loss, both to the school of which he was a part, and to the profession at large. Dr. S. was also one of the physicians to the New York Hospital. He has acquired an enviable reputation in the diagnosis and treatment of thoracic diseases. H.

